# Behavioral Interference by Emotional Stimuli: Sequential Modulation by Perceptual Conditions but Not by Emotional Primes

**DOI:** 10.3390/vision9030066

**Published:** 2025-08-01

**Authors:** Andrea De Cesarei, Virginia Tronelli, Serena Mastria, Vera Ferrari, Maurizio Codispoti

**Affiliations:** 1 Department of Psychology, University of Bologna, Viale Berti Pichat, 5, 40127 Bologna, Italy; virginia.tronelli2@unibo.it (V.T.); serena.mastria4@unibo.it (S.M.); maurizio.codispoti@unibo.it (M.C.); 2 Department of Medicine and Surgery, University of Parma, Via Volturno, 39, 43125 Parma, Italy; vera.ferrari@unipr.it

**Keywords:** emotional interference, spatial frequencies, cognitive control

## Abstract

Previous studies observed that emotional scenes, presented as distractors, capture attention and interfere with an ongoing task. This behavioral interference has been shown to be elicited by the semantic rather than by the perceptual properties of a scene, as it resisted the application of low-pass spatial frequency filters. Some studies observed that the visual system can adapt to perceptual conditions; however, little is known concerning whether attentional capture by emotional stimuli can also be modulated by the sequential repetition of viewing conditions or of emotional content. In the present study, we asked participants to perform a parity task while viewing irrelevant natural scenes, which could be either emotional or neutral. These scenes could be either blurred (low-pass filter) or perceptually intact, and the order of presentation was balanced to study the effects of sequential repetition of perceptual conditions. The results indicate that affective modulation was most pronounced when the same viewing condition (either intact or blurred) was repeated, with faster responses when perceptual conditions were repeated for neutral distractors, but to a lesser extent for emotional ones. These data suggest that emotional interference in an attentional task can be modulated by serial sensitization in the processing of spatial frequencies.

## 1. Introduction

Emotional events (both appetitive and aversive ones) engage motivational systems and result in a modulation of subjective state, physiological activities, behavior, and cognitive processes [1,2,3]. When an emotional event happens, attentional resources are diverted to facilitate its processing. In particular, when we are focused on an activity and an irrelevant emotional event occurs, attention is diverted to the emotional event, resulting in a slowing of response times (emotional interference). Several studies have documented emotional interference in visual and cross-modal tasks [4,5,6,7,8,9,10,11,12]. Although allocation of attention to emotional stimuli in these studies was non-voluntary, other studies observed that emotional interference was modulated by contextual factors, indicating that it cannot be considered a fully automatic process [9,13,14]. More specifically, emotional interference, assessed during a parity judgment task through the presentation of visual distractors (emotional and neutral natural scenes), was reduced by picture repetition [15]. Similarly, the frequency of presentation of distractors (emotional pictures) has been shown to modulate emotional interference in a perceptual task [8].

In terms of visual processing, response to emotional natural scenes depends upon understanding and evaluation of the visual input [16,17,18,19,20]. Several studies investigated the role of picture composition on identification and categorization, and emphasized the contribution of spatial frequencies (SFs) to scene understanding. Spatial frequencies refer to the number of times a detail is repeated in a unit of space (e.g., a degree of visual angle or the whole object), and continuously span from low spatial frequency, i.e., large details encompassing large areas of a picture, to high spatial frequencies, i.e., tinier details in the picture space. The whole range of spatial frequencies contributes to picture identification, and optimal understanding of the visual input is achieved when the least extreme (i.e., neither the lowest nor the highest) parts of the spatial frequency range are preserved [21,22,23]. Moreover, the use of spatial frequencies has been shown to be modulated by learning [24,25], beginning from the earliest stages of visual processing [26]. Upon repeated experience with spatial frequency ranges or with tasks that focus on a SF range, the processing of spatial frequencies can be facilitated. In particular, it has been shown that repeated exposure to a sensory set (e.g., [27]), or performance in tasks for which some kind of information is diagnostic (e.g., [28]), facilitates the processing of repeated or diagnostic information. Similarly, SF processing can be attentionally primed [29]. Altogether, these data indicate that SF processing can be modulated by both sustained experience over long training sessions (SF sensitization, perceptual learning, and perceptual set) and by trial-to-trial phasic priming of SF processing (serial adaptation, see [30]).

Previous studies asked to what extent emotional processing is modulated by the perceptual properties of the pictures, and, in particular, by SFs, at the subjective, behavioral, and electrocortical levels. Specifically, several studies have degraded emotional and neutral scenes, showing that emotional processing proceeds together with picture identification [16,17,18,19,23,31,32]. For instance, no difference in emotional modulation of electrocortical activity was observed for high- and low-spatial frequency filtered emotional pictures once they were equated for identification [31]. Finally, a previous study that presented low-pass filtered emotional pictures while asking participants to perform a cross-modal tone categorization task observed no difference in emotional interference between intact pictures and mildly blurred (59 cycles/image) pictures [6].

In cognitive tasks, interference can be counteracted by cognitive control, which has been suggested to underlie sequential effects in conflict tasks. It has been observed since the 90s [33] that interference effects (incongruent–congruent differences) are reduced, absent, or even inverted after an incongruent trial compared with a congruent trial (congruence sequence effect, CSE). While early models ascribed this phenomenon to the engagement of processes that are devoted to reducing conflict (conflict-adaptation models; e.g., [34]), later models noted that the repetition of stimuli or features across trials led to a CSE pattern of results [35]. More recently, these two views have been integrated, suggesting that episodic event representations are formed at every trial, based on the binding of sensory/perceptual, motor, and control components of each trial. If some of these components are repeated with respect to one or more previous trials, the previous cognitive set is retrieved [36,37,38,39] and may facilitate the processing of the current trial. For instance, if the previous trial was an incongruent condition which may prompt cognitive inhibition (e.g., an incongruent Stroop trial), and a trial property (e.g., color) is repeated in the actual trial, then inhibitory control processes are also retrieved and eventually facilitate performance in the current trial by reducing interference [39]. Therefore, binding-retrieval mechanisms have been suggested to underlie sequential cognitive control effects [35,38,39].

Research on sequential cognitive control and emotional processing showed mixed results. Previous studies, using the additional singleton paradigm [40], manipulated the repetition of target color between the previous and the actual trial and the reward given in the previous trial [41,42]. These studies observed that the repetition (vs. swap) of the singleton target color modulated response times depending on the reward in the previous trial, with facilitation following color repetition for previously high-gain trials and slowing following color swap for high-gain trials. Altogether, these studies suggest that binding-retrieval effects may be modulated by emotion. Conversely, other studies that examined interference by task-irrelevant factors (incongruency, non-correspondence) manipulated arousal in the previous trial (or in the intertrial interval, ITI), and failed to observe any interference reduction as a function of the previous relevance (presentation of an accessory stimulus in the pre-stimulus interval [43]; novelty of the previous trial [44]; presentation of emotional pictures during the ITI [45]).

Taken together, the reviewed studies suggest that sequential effects may affect interference in the current trial either through a modulation of the bottom-up processing of visual scenes or as a consequence of the repetition of emotional valence. Consistently, the aim of the present study is to examine the degree to which emotional interference in an attentional task (parity judgment) is sequentially modulated by emotion and by the perceptual aspect of pictures. To this end, we devised a parity task that replicated that of Codispoti et al. [15], and presented two digits on the sides of the monitor. These two digits could be both odd or even, or one odd and one even. The task of participants was to indicate whether they had the same parity (e.g., 2 and 8) or a different parity (e.g., 1 and 8). Together with the numbers, a picture was presented in the center of the monitor, which could be emotional (pleasant or unpleasant) or neutral, and presented in its intact form or in a blurred (low-pass filtered) version. In a balanced design, to investigate sequential modulation of emotional interference, we manipulated both the emotional relevance and the level of degradation of central distractor in the current, as well as in the previous trial.

In terms of results, it is expected that the content of the irrelevant pictures will interfere with the parity task, similarly for blurred and non-blurred pictures [6]. Concerning the role of emotional value and viewing conditions of distractors in the present and previous trials, two opposite scenarios can be expected. First, if both emotional value and viewing condition are stored in episodic representations of the previous trial, then they should concur to modulate performance upon retrieval, resulting in a reduction in emotional interference upon repetition of either property. On the other hand, it is possible that repeated presentations of distractor pictures of the same perceptual quality (intact or blurry) may establish a perceptual set that aids the processing of distractor pictures, eventually resulting in a more pronounced emotional interference upon repetition of degradation level.

## 2. Method

### 2.1. Participants

A total of 55 individuals, including university students and adult workers, took part in this study. After providing their informed consent, participants were required to confirm they were of legal age. The study adhered to the ethical guidelines outlined in the Declaration of Helsinki and received approval from the University of Bologna’s Ethics Committee. To determine the necessary sample size for analyzing the four-way interaction of interest (Current Emotion × Previous Emotion × Current Filter × Previous Filter), a power analysis was conducted. The parameters included α = .05, power = .80, partial eta squared (η^2^_p_) = .1 [46], and a correlation of .24 among repeated measures calculated on an independent sample of participants. The results indicated that a minimum of 20 participants were required for the study.

### 2.2. Stimuli

The target stimuli consisted of digits ranging from 1 to 9. For the distractor images, a total of 296 pictures were sourced from the International Affective Picture System (IAPS; [47]), as well as public-domain pictures available on the Internet. The picture set was composed of 148 emotionally arousing images (equally divided into pleasant and unpleasant) and 148 neutral natural scenes, of 383 horizontal × 287 vertical pixels in size. Each image was available in both its original form (i.e., intact images) and a degraded version (i.e., blurred images). Blurred pictures were obtained through a low-pass filter whose function passed all spatial frequencies below a falloff value of 13.45 cpi, and declined parabolically to a cutoff value of 40.36, above which all frequencies were filtered out [17,21,23,31]. Each participant saw each picture in only one condition, either intact or blurred. Additionally, four extra images were included exclusively for the practice phase.

### 2.3. Procedure

**Figure 1:**
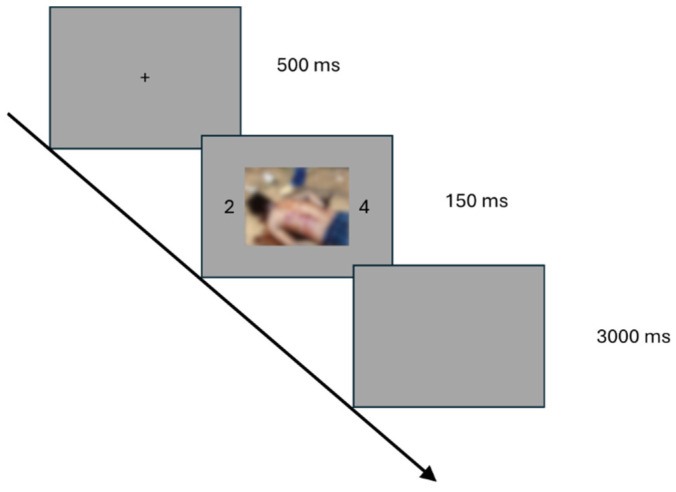
Trial procedure. An example of a trial procedure in which a blurred unpleasant emotional image is shown.

The experiment was programmed on OpenSesame version 4.0.2 [48] and was conducted online using the Jatos platform [49]. Prior to the task, participants completed four practice trials to become familiar with the procedure. Each trial started with a fixation point displayed for 500 milliseconds, followed by a brief presentation of two numbers with a picture placed between them for 150 milliseconds. On a 24″ monitor placed at 60 cm from the participant, number size would be 0.85 horizontal × 1.69 vertical degrees of visual angle, placed apart (center-to-center) by 26°. Picture size would be 20.12 horizontal × 15.16 vertical degrees of visual angle, and space between the outer sides of the picture and the inner sides of the number would be 1.69°. The pattern containing the numbers and the picture then disappeared and was followed by a blank screen lasting 3000 milliseconds. An illustration of the experimental procedure can be seen in Figure 1. Participants were instructed to classify the number pairs based on their parity—determining whether both were either even or odd (same parity, e.g., 2 and 8) or whether one was even and the other odd (different parity, e.g., 1 and 8). They were told to ignore the central image, which could appear either in its original or blurred version along with the digits. Responses were made using the Z and M keys on a QWERTY keyboard, with the key assignment counterbalanced across participants. The experiment consisted of two blocks, each containing 148 trials. To control for visual similarity effects, certain number combinations were restricted: visually similar pairs (e.g., 3–8, 6–9, 1–7) were avoided, numbers within a pair were never repeated (i.e., if the current trial contained the pair 2 and 8, the following trial would neither contain 2 nor 8), and numbers differing by only one digit (i.e., 1 and 2) were never paired together. Additionally, several constraints were applied to trial sequencing: No more than six consecutive trials featured the same emotional content (emotional or neutral), no more than six consecutive trials alternated between emotional categories, no more than six successive trials had the same level of image degradation, and no more than seven consecutive trials varied in degradation levels. The entire experiment lasted approximately 30 min.

### 2.4. Analysis

Practice trials, the first trial of each block, trials with reaction times (RTs) exceeding 2.5 standard deviations (SD) from the individual mean, or in which an inaccurate response was given, as well as trials that followed an error (to avoid post-error slowing and reduction in interference [50,51]), were excluded from the RT analyses. After cleaning the RTs, participants who remained with less than 50% of the trials were excluded (N = 6), leaving a sample of 49 participants for the analysis. Mean RT data were analyzed with repeated-measures ANOVA, with the following within-subject factors: Current Emotion (2 levels: Emotional and Neutral), Current Filter (2: Intact and Blurred), Previous Filter (2 levels: Previous Intact and Previous Blurred), and Previous Emotion (2 levels: Previous Emotional and Previous Neutral). When necessary, Huynh–Feldt correction was applied to adjust for violations of sphericity. Additionally, partial eta squared (η^2^_p_) values were computed and reported to indicate effect sizes.

## 3. Results

### 3.1. Effects of Current Emotion

**Figure 2:**
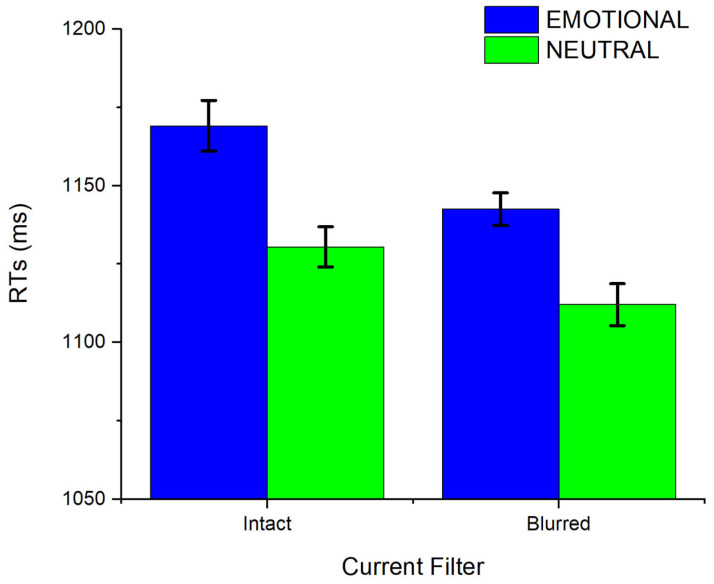
Mean RTs for emotional and neutral stimuli as a function of Current Filter. Error bars reflect ±1 within-subject standard errors of the mean [52].

Response times are shown in Figure 2. A significant main effect of Current Emotion was observed, F(1, 48) = 18.50, *p* < .001, η^2^_p_ = .28. Responses to emotional images were slower compared to neutral images (M = 1155.87 ms, SD = 257.93; M = 1121.22 ms, SD = 235.59, respectively). Moreover, the interaction between Current Emotion and Current Filter was not significant, F(1, 48)= 0.35, *p* = .559, η^2^_p_ = .01, and a significant effect of Current Emotion was observed both for intact and for blurred pictures, F(1, 48) = 12.20, *p* = .001, η^2^_p_ = .20 and F(1, 48) = 13.17, *p* = .001, η^2^_p_ = .22, respectively. A significant effect of Current Filter was also observed, F(1, 48) = 13.46, *p* < .001, η^2^_p_ = .22, with slower reaction times when distractors were non-blurred compared to when they were blurred (M = 1149.41 ms, SD = 254.62; M = 1127.00 ms, SD = 237.60, respectively).

### 3.2. Effects of Previous Emotion and Filter

**Figure 3:**
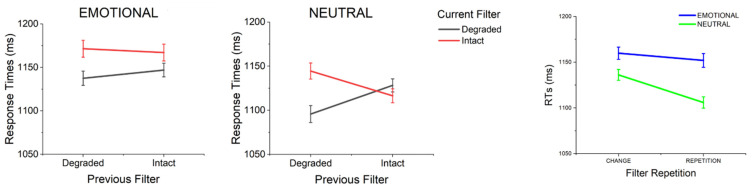
Left and center panels: Mean RTs for emotional and neutral stimuli as a function of Current and Previous Filter. Right panel: Mean RTs for emotional and neutral stimuli as a function of filter change/repetition. Error bars reflect ±1 within-subject standard errors of the mean [52].

A significant three-way interaction was observed between Current Emotion, Current Filter, and Previous Filter, F(1, 48) = 4.10, *p* = .048, η^2^_p_ = .08, as illustrated in Figure 3. Separately analyzing emotional and neutral trials, we observed that in emotional trials only the main effect of Current Filter was significant, F(1, 48) = 9.35, *p* = .004, η^2^_p_ = .16, with slower reaction times when distractors were intact compared to when they were blurred (M = 1168.81 ms, SD = 268.29, and M = 1142.74 ms, SD = 251.61, respectively), and no other main effect or interaction was significant, Fs(1, 48) < 1.65, *p*s > .21, η^2^_p_s < .03. For neutral trials, a main effect of Current Filter was observed, F(1, 48) = 5.59, *p* = .02, η^2^_p_ = .10, along with a significant interaction between Current and Previous Filter, F(1, 48) = 20.04, *p* < .001, η^2^_p_ = .30. Breaking this interaction with the Current Filter, we observed that currently degraded trials that were also degraded in the previous trial were significantly faster compared to currently degraded trials that were intact in the previous trial, F(1, 48) = 8.48, *p* = .005, η^2^_p_ = .15. Conversely, currently intact stimuli that were preceded by intact stimuli were faster than currently intact stimuli that were preceded by degraded trials, F(1, 48) = 6.31, *p* = .015, η^2^_p_ = .12. For neutral stimuli that were degraded in the previous trial, faster responses were observed if stimuli were also degraded (vs. intact) in the current trial, F(1, 48) = 17.22, *p* < .001, η^2^_p_ = .26. For neutral stimuli that were presented intact in the previous trial, no significant difference depending on the viewing condition in the actual trial was observed, F(1, 48) = 1.57, *p* = .22, η^2^_p_ = .03. Following the significant three-way interaction, a significant interaction between the Current and Previous Filter was observed, F(1, 48) = 11.70, *p* < .001, η^2^_p_ = .20, showing that participants responded more quickly when the filter condition was repeated rather than changed (M = 1128.54 ms, SD = 243.20, and M = 1147.66 ms, SD = 249.33, respectively). The main effects of Previous Emotion and Previous Filter were not significant, F(1, 48) = 0.003, *p* = .954, η^2^_p_ < .001 and F(1, 48) = 0.20, *p* = .654, η^2^_p_ = .004, respectively.

Finally, the interaction between Current Emotion and Previous Emotion was not significant, F(1, 48) = 2.17, *p* = .148, η^2^_p_ = .04, and significant effects of Current Emotion were observed following both emotional trials, F(1, 48) = 18.13, *p* < .001, η^2^_p_ = .27, and neutral trials, F(1, 48) = 6.78, *p* = .012, η^2^_p_ = .12. No other significant interactions were observed in these analyses, Fs ≤ 1.667, *p*s ≥ .203, η^2^_p_ s ≤ .034.

### 3.3. Further Analysis: Filter Repetition/Change

Following the significant interaction between Current Emotion, Current Filter, and Previous Filter, which indicated that emotional interference varied with the repetition or change in picture degradation, we recoded trials to understand whether changes in emotional interference varied on a trial-by-trial basis, or increased with repetitions (regardless of the type of perceptual degradation). Therefore, we coded trials as change trials, i.e., when picture degradation changed from the N^th^ trial to the preceding (N-1) one, or as repetition trials, which were further divided in one repetition (same perceptual condition in trials N and N-1, but different in N-2), two repetitions (same perceptual condition in trials N, N-1 and N-2, but different in N-3), or three or more repetitions (same perceptual condition in trials N, N-1, N-2 and N-3). Then, we submitted these data to an ANOVA with factors Current Emotion (two levels: emotional vs. neutral) and Repetitions (four levels: change, one repetition, two repetitions, three or more repetitions).

**Figure 4:**
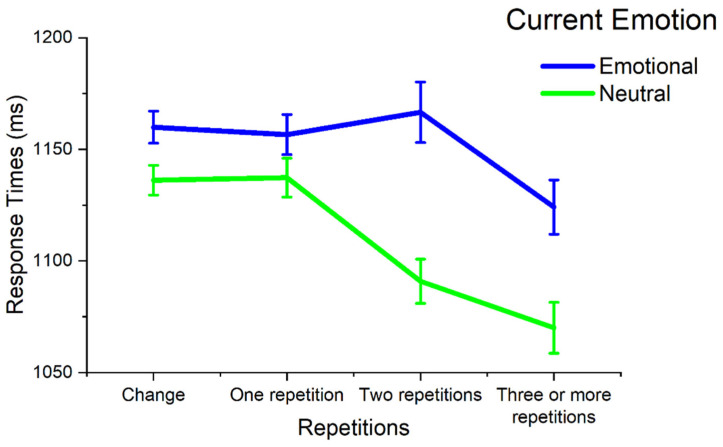
The effects of the number of perceptual repetitions and current emotion on Mean RTs. Error bars reflect ±1 within-subject standard errors of the mean [52].

A significant main effect of Current Emotion was observed, F(1, 48) = 20.94, *p* < .001, η^2^_p_ = .30, indicating slower responses to emotional compared with neutral stimuli, along with a significant main effect of Repetitions, F(3, 144) = 12.76, *p* < .001, η^2^_p_ = .21, indicating that response times were faster with repetitions, linear contrast F(1, 48) = 33.50, *p* < .001, η^2^_p_ = .41. Moreover, a significant interaction between Current Emotion and Repetitions was observed (Figure 4), F(3, 144) = 4.00, *p* = .01, η^2^_p_ = .08, with emotional interference increasing with repetitions (change trials, M = 23.74, SD = 66.44; 1 repetition, M = 19.29, SD = 87.26; 2 repetitions, M = 75.70, SD = 121.58; 3 or more repetitions, M = 54.05, SD = 129.16). Following this significant interaction, the effects of Current Emotion were separately assessed at each repetition level. For change trials, a significant effect of Current Emotion was observed, F(1, 48) = 6.25, *p* = .016, η^2^_p_ = .12. For repetition trials, no significant modulation was observed for one repetition, F(1, 48) = 2.40, *p* = .128, η^2^_p_ = .05, but a significant modulation was observed for two repetitions, F(1, 48) = 19.00, *p* < .001, η^2^_p_ = .28, and for three or more repetitions, F(1, 48) = 8.58, *p* = .005, η^2^_p_ = .15. A significant effect of Repetitions was observed both on emotional and neutral trials, F(3, 144) = 3.40, *p* = .033, η^2^_p_ = .07, and F(3, 144) = 15.71, *p* < .001, η^2^_p_ = .25, respectively. For emotional pictures, faster responses were observed for three or more repetitions compared with all other conditions, Fs(1, 48) > 5.84, *p*s < .02, η^2^_p_s > .11, and no difference between all other conditions, Fs(1, 48) < 0.34, *p*s > .562, η^2^_p_s < .01. For neutral pictures, significant differences were observed between all conditions, Fs(1, 48) > 11.98, *p*s < .001, η^2^_p_s > .20, except between change and one repetition, F(1, 48) = 0.02, *p* = .894, η^2^_p_ < .001, and between two and three or more repetitions, F(1, 48) = 3.07, *p* = .086, η^2^_p_ = .06.

The same analysis was carried out to investigate the sequential effect of valence repetition, analyzing the effects of Current Emotion as a function of the change in valence, or of the repetition of the same valence for one, two, or three or more trials (four levels: change, one repetition, two repetitions, three or more repetitions). No significant interaction was observed in this analysis, F(3, 144) = 2.28, *p* = .10, η^2^_p_ = .05.

## 4. Discussion

Here, we examined emotional interference in a parity judgment task and focused on the sequential effects of emotional value and degradation level of distractors. Consistent with previous research, we observed emotional modulation of response times, with slower responses for emotional compared with neutral distractors [4,5,6,7,8,9,10,11,12,15]. Moreover, emotional interference did not vary with picture perceptual composition (intact vs. blurring), consistent with a previous study that manipulated the level of degradation of emotional pictures occurring during an auditory discrimination task (high vs. low pitch, [6]). Here and in previous studies, a low-pass filter of 40 cpi was associated with optimal scene understanding, both in terms of semantic tasks (naming [23]), categorization [21], and gist understanding [17,21,31]. Therefore, the present results are consistent in suggesting that, even in the presence of picture degradation, attention is diverted from the current task upon identification of the emotional value of a distractor picture.

Although picture degradation did not modulate emotional interference, we observed a significant sequential effect that occurred when the same picture quality condition (intact or blurred) was repeated consecutively in multiple trials. Interestingly, this effect was evident for neutral distractors that prompted faster reaction times the more the same degradation condition was repeated, while this occurred to a lesser extent for emotional stimuli (only after three or more repetitions of the same degradation condition).

This result seems to reflect visual system adaptation to the spatial frequency range that is currently being displayed (e.g., the whole frequency range in the case of intact pictures, and ≤40 cpi in the case of blurred pictures), consistent with SF processing facilitation or preference that has been observed in previous studies [24,27,28,29]. However, the present data seem to indicate that this sensitization does not require a long training session but rather can be observed after a few trials. Even more interestingly, the observation that this effect happens at a slower pace for emotional compared with neutral stimuli suggests that emotional stimuli presentation counteracts the benefits of repeated presentation of the same degradation condition. This result is in line with previous studies indicating that emotional processing and interference are modulated by top-down control [8,9,14,15]. Moreover, it suggests that the processing of emotional distractors may benefit less from sensitization, possibly because of a “better safe than sorry” strategy, according to which it is more valuable to process emotional stimuli even when these result in behavioral interference.

Previous studies that observed the perception of objects or properties being primed by the immediately preceding items described this effect as a serial dependence effect and questioned whether serial dependence effects originate more from perceptual or decisional processes [30]. However, studies that investigate serial dependence mainly focused on explicit judgements, while here no explicit processing of distractor pictures was required. Therefore, while future studies might further explore the extent to which sequential processing of spatial frequencies reflects an attentional or a perceptual phenomenon, here it can be excluded that an explicit judgment task may underlie this serial effect.

Finally, we did not observe that the emotional value of the distractor in the previous trial modulated emotional interference in the current trial. This result is consistent with previous studies, which examined congruency sequence effects (CSE) and investigated the role of relevance of the previous trial, operationalized as emotional relevance [45], novelty [44], or presence of a task-irrelevant accessory stimulus [43]. Similarly, studies that examined electrocortical responses (early and late components of Event-Related Potentials, ERPs) to emotional and neutral pictures observed similar affective modulation for trials preceded by either neutral or emotional pictures [53,54]. On the other hand, other studies observed that reward-associated items are better maintained in visual working memory [55,56,57,58], and result in priming of spatial attention in the subsequent trial (reward priming [41,42]; see also [59,60]). Interestingly, studies that observed modulation of sequential effects by the relevance of the previous trial adopted a visuo-spatial attention paradigm (singleton paradigm [40]), while studies that did not observe modulation of sequential effects by the relevance of the previous trial examined tasks that built on visuo-motor correspondence (Simon task [61]) or semantic/arithmetic judgements (parity task [62]). Therefore, building on the observation that cognitive control is a multi-faceted construct that includes diverse processes related to, e.g., attention, working memory, and decision-making [63,64], one possibility is that the degree of modulation by pre-stimulus relevance depends on the overlap between pre-stimulus relevance information and the type of resources required by the task. In sequential singleton paradigms [41,42,59,60,65], reward in the previous trial, as well as target and distractor information in the current trial, are visually presented and compete for visual attention resources. In other tasks, however (e.g., Simon and parity judgements), interference in the current trial happens at the sensory/motor (Simon) or semantic/arithmetic (parity) level. Therefore, one issue that might be examined by future studies is the relationship between pre-stimulus relevance, processing level at which interference happens, and emotional modulation of sequential effects.

## 5. Conclusions

Here, we examined sequential modulation of emotional interference. While no sequential effects that dampened interference were observed, neither by perceptual properties (degradation) nor by previous distractor value, emotional interference was enhanced by repetition of the same perceptual conditions, and this was due to a more pronounced effect of repeated presentations of the same degradation level on neutral, as compared with emotional distractors. The present results indicate that the emotional value of distractors interferes both with a parity task and with the capability to adapt to repeated perceptual presentation conditions.

## Data Availability

Data available in a publicly accessible repository at https://osf.io/b6jsa (accessed on 16 July 2025).
